# The study of consciousness is an integral part of the Indian heritage

**DOI:** 10.4103/0019-5545.49461

**Published:** 2009

**Authors:** M. R. Asha

**Affiliations:** Reiki Practitioner & Freelance writer, 788/160, 18th Cross, Ramanuja Road, Mysore. E-mail: ambleewine@yahoo.co.in

Sir,

This is in response to the article entitled “The Conscious Access hypothesis: Explaining the Consciousness” published in IJP, Vol 50, Issue 1, Jan-Mar. 2008.

The concept of consciousness is a much debated issue, globally. People have their own definitions, theories based on their experiences and perceptions. Your plea for deeper research in this area is perfectly timed.

I strongly believe that possibilities of helping people heal (NOT JUST FOR ACADEMIC INTEREST) themselves through lesser known (scientifically) but very effective approaches should be explored. Naturally, it follows that we try simpler, closer-to-nature methods that align elegantly with our inner divine design.

Being a practitioner of Reiki, a form of energy medicine and a healing path, I've conducted some experiments (involving Reiki) on water and Porcine DNA with interesting results, where changes in “Absorption spectra” and pH were observed. Speaking from my personal experience, there have been plenty of incidents (Believe me, they are NOT PLACEBO effects!) which are difficult to explain in terms of what CURRENT SCIENCE believes. Published literature on Reiki is sparse. Nevertheless, many laudable attempts have been made, of late, to understand the mechanisms underlying healing at cellular and sub-cellular levels.

James Oshman, one of the leading authorities on the scientific understanding of hands-on healing and author of “Energy Medicine: The Scientific Basis” opines that the integration of research science with complementary medicine is a tremendously exciting endeavor.[1] In elaborating the application of energy medicine, he explains that, from the scientific perspective, the higher intelligence (that we refer to as consciousness) is nothing but the innate intuitive inner wisdom we all possess and can access when we relax our mental processes (diminishing the activity of conscious mind that essentially occurs during meditation and many other healing modalities) and allow our subconscious to sort out what is going on. He hypothesizes that, tissue that is damaged or diseased, emits signals that are induced into the energy systems of the hands of Reiki practitioners that serve to guide them to the right places. Behaving like “Liquid Crystals”, living tissues are composed of semiconductor materials which form a sophisticated electronic circuit. That's how different frequencies can be both projected and sensed. He also touches upon the non-locality aspects of consciousness using some concepts from quantum physics.

Following are some exciting possibilities that can be explored in future.

Effect of Reiki (or any very low frequency electromagnetic wave) on

1. Immunity (measuring Ig A in human saliva)
2. Cell Membrane permeability- (which can have a HUGE IMPACT on so many areas, including Blood-brain-barrier)
3. Endorphin levels and neurotransmitter levels
4. Epigenetics (by way of uncoiling and exposing hitherto unexplored segments of DNA)

If enough people on similar wavelength (hopefully achieving the “Critical mass”!) team up, I'm positive, we can achieve lots and lots.
